# Assessing the impact of a motivational intervention to improve the working lives of maternity healthcare workers: a quantitative and qualitative evaluation of a feasibility study in Malawi

**DOI:** 10.1186/s40814-021-00774-7

**Published:** 2021-01-29

**Authors:** Abi Merriel, Zione Dembo, Julia Hussein, Michael Larkin, Allan Mchenga, Aurelio Tobias, Mark Lough, Address Malata, Charles Makwenda, Arri Coomarasamy

**Affiliations:** 1 Institute for Metabolism and Systems Research, University of Birmingham, Edgbaston, Birmingham, B15 2TT UK; 2 Population Health Sciences, Bristol Medical School, University of Bristol, Bristol, UK; 3 Parent and Child Health Initiative, Lilongwe, Malawi; 4 Independent Maternal Health Consultant, Aberdeen, UK; 5 Department of Psychology, Aston University, Birmingham, UK; 6 Independent Psychotherapist and Organisational Consultant, Aberdeen, UK; 7 Malawi University of Science and Technology, Thyolo, Malawi

**Keywords:** Appreciative Inquiry, Patient satisfaction, Staff working life, Malawi, Maternity care

## Abstract

**Background:**

Globally too many mothers and babies die during childbirth; 98% of maternal deaths are avoidable. Skilled clinicians can reduce these deaths; however, there is a world-wide shortage of maternity healthcare workers. Malawi has enough to deliver 20% of its maternity care. A motivating work environment is important for healthcare worker retention. To inform a future trial, we aimed to assess the feasibility of implementing a motivational intervention (Appreciative Inquiry) to improve the working lives of maternity healthcare workers and patient satisfaction in Malawi.

**Methods:**

Three government hospitals participated over 1 year. Its effectiveness was assessed through: a monthly longitudinal survey of working life using psychometrically validated instruments (basic psychological needs, job satisfaction and work-related quality of life); a before and after questionnaire of patient satisfaction using a patient satisfaction tool validated in low-income settings with a maximum score of 80; and a qualitative template analysis encompassing ethnographic data, semi-structured interviews and focus groups with staff.

**Results:**

The intervention was attended by all 145 eligible staff, who also participated in the longitudinal study. The general trend was an increase in the scores for each scale except for the basic psychological needs score in one site. Only one site demonstrated strong evidence for the intervention working in the work-related quality of life scales. Pre-intervention, 162 postnatal women completed the questionnaire; post-intervention, 191 postnatal women participated. Patient satisfaction rose in all three sites; referral hospital 4.41 rise (95% CI 1.89 to 6.95), district hospital 10.22 (95% CI 7.38 to 13.07) and community hospital 13.02 (95% CI 10.48 to 15.57). The qualitative data revealed that staff felt happier, that their skills (especially communication) had improved, behaviour had changed and systems had developed.

**Conclusions:**

We have shown that it is possible to implement Appreciative Inquiry in government facilities in Malawi, which has the potential to change the way staff work and improve patient satisfaction. The mixed methods approach revealed important findings including the importance of staff relationships. We have identified clear implementation elements that will be important to measure in a future trial such as implementation fidelity and inter-personal relationship factors.

**Supplementary Information:**

The online version contains supplementary material available at 10.1186/s40814-021-00774-7.

## Introduction

Globally, 295,000 mothers die [1], 2.5 million babies die in the first week of life [2] and 2.6 million are stillborn [3] every year. High-quality skilled care directly mediates the morbidity and mortality of mothers and neonates [4, 5]. Skilled care could prevent 98% of maternal deaths worldwide [4]. Globally, there are too few skilled clinicians to deliver this care [6–8], leading to inadequate care, demotivation, dissatisfaction and burnout, and subsequently poor retention [9]. Increasing staffing and adequate renumeration can improve retention, but it is not enough. Positive personal factors such as improved interest, enjoyment and satisfaction can motivate the workforce, and motivation is an important factor in staff retention [10, 11].

Malawi is a low-income country with a high maternal and neonatal mortality rate [1, 2]. Although there are many contributing factors [12], Malawi has a significant shortage of staff, with only enough maternity healthcare workers (MHCWs) to deliver 20% of their maternity care [7]. Recruitment and retention of MHCWs is therefore vital.

**Fig. 1:**
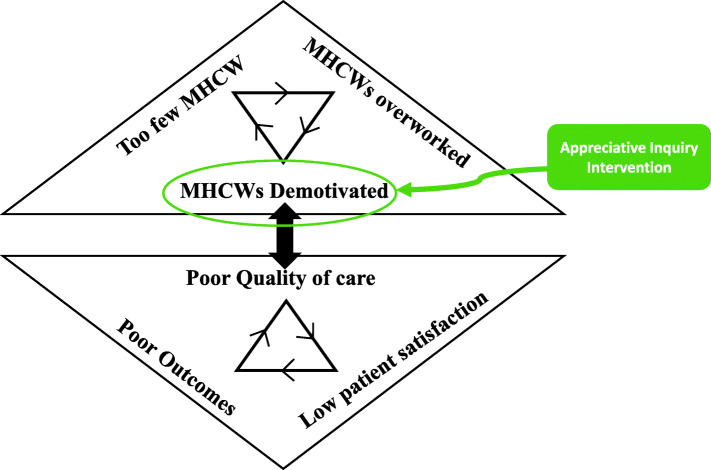
Theory of change

We developed a rudimentary theory of change relating to quality of care and motivation levels of healthcare workers (Fig. 1). We postulated that there was a self-perpetuating cycle of too few MHCWs, who became overworked leading to demotivation, which served to make work unattractive, thus reinforcing the cycle. This is directly linked to delivery of poor quality of care, leading to unsatisfied patients and poor outcomes, all of which feedback to further demotivate MHCWs. Possible methods for improving working-life and therefore motivation include improving supervision, promotion opportunities, and collaborative standard setting [13]. Historically, there has been a focus on improving an individual’s knowledge and skills; however, the evidence for this altering clinical outcomes is weak [13]. In Malawi, a negative association with psychological wellbeing has been found with attending a training course [14]. Supportive supervision can increase job satisfaction and motivation; however, it has little effect on clinical outcomes and it requires significant external support [15]. Staff motivation has been increased using quality improvement and patient safety activities [16], suggesting that staff are motivated by providing good care. Findings from our study of working lives support this [17]. The role of interpersonal relationships seems to be important to improve psychological wellbeing in Malawi [14, 17]. We therefore decided that an organisational approach would allow us to be non-punitive; instead of suggesting ‘you’re not doing this well’, we could ask ‘how can the system change to...’

**Fig. 2:**
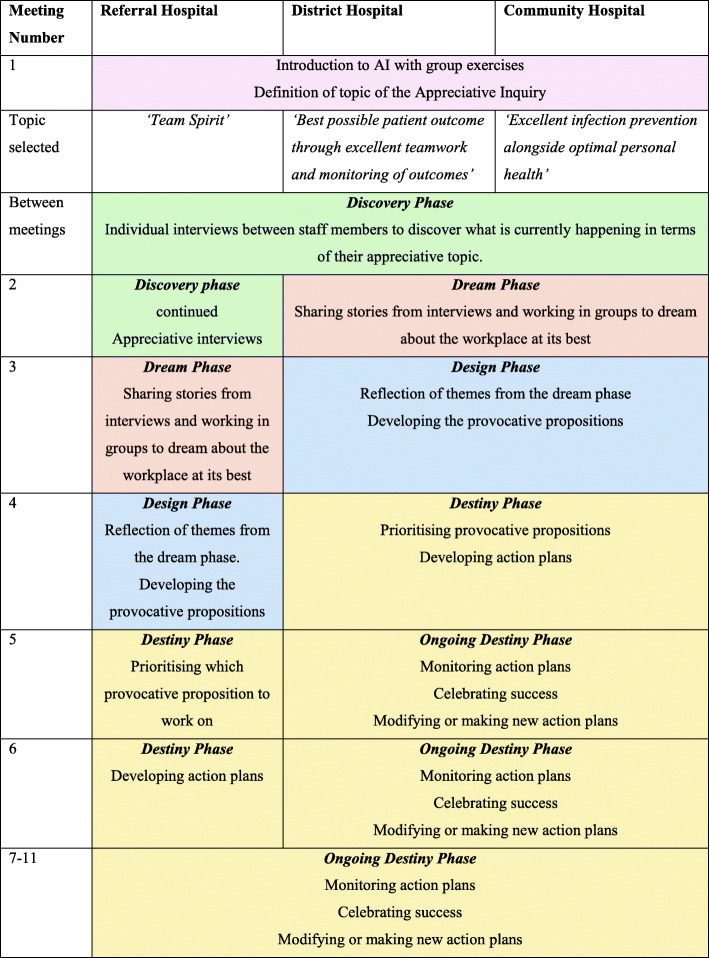
Summary of the AI intervention at each site

We identified Appreciative Inquiry (AI) as a motivational intervention fitting with our goals [18]. AI’s premise is that every organisation has something within it that works well and can be built on [18]. AI is an organisational change action cycle with four phases: ‘discover, dream, design and destiny’ [18]. It brings people together in ‘summits’ to go through the phases [18]. It has been widely used in the private sector resulting in increased productivity and workforce retention [18]. However, high-quality empirical evidence for AI is lacking [19], and this is especially true for clinical environments. In healthcare, targets for the intervention have included changing work practices, improving the work environment and exploring professional development initiatives [20]. The only randomised trial evidence of AI changing clinical care was in primary care in South Africa [21]. The remainder of the evidence we have identified from low-income settings is from maternity care based in Nepal [22] and India [23, 24], which are very different cultural contexts to Malawi. To enable AI to be successfully implemented in a low-cost way in Malawi, we decided to adapt AI. AI is usually carried out in a series of large meetings, often referred to as ‘summits’ where all relevant team members(e.g. the whole maternity department) would be invited to participate at a particular time (e.g. several days) or perhaps series of times (e.g. half days). Due to the need to deliver this intervention in a practical way both from a clinical care and resource perspective, we decided to modify AI from its original summit methodology into multiple significantly shorter (1–2 h) meetings (Fig. 2 illustrates the intervention).

We aimed to assess the feasibility of conducting a future trial of a modified AI intervention to improve maternity care through firstly understanding whether it was possible to implement the adapted intervention into the working environment and if staff would/could participate. Secondly, to test data collection procedures and understand whether the modified intervention could harness the positive ethos of AI and therefore have the potential to foster organisational change and improve working lives of staff and satisfaction for patients.

## Methods

### Setting

Three government facilities in the Central Region of Malawi were selected in conjunction with the local research teams and District Health Officers. The sites were selected to represent different parts of the government health system in Malawi. In addition to this, there had to be enough staff to enable an action cycle approach and enough deliveries for there to be dedicated MHCWs. This meant that small health facilities with just a few hundred births per year and two or three staff working there were not included in this study. The study lasted 1 year from July 2015.

Study sites were the high-risk postnatal ward of a district referral hospital (RH) (15,000 deliveries per year); labour and postnatal wards of a district hospital (DH) (3700 deliveries per year) and maternity wards of a community hospital (CH) (4500 deliveries per year).

All MHCWs at the study sites were invited to participate, including nurses, auxiliary staff and clinical officers. As we were aiming to be motivational, we were inclusive and did not require staff to attend every session, instead they were invited to attend those they could (e.g. they were encouraged not to attend if on Annual Leave or working night duty), furthermore, some staff who were on clinical duty had to remain on the wards to treat patients. This was actively encouraged, and they were included in the refreshments and a modest transport allowance provided for those participating in the intervention, to avoid staff leaving the ward to attend the session for these small benefits.

### Methodologies

We used quantitative and qualitative methods in parallel to assess the feasibility of this intervention for a future study. We did not use the findings from one method to inform the study design of the other [25]. To support the reporting of this, we have incorporated elements from the ‘Good Reporting of Mixed Methods Studies’ guidelines [24], and also drawn on the CONSORT statement extension to randomised pilot and feasibility trials [26]. We believed it was necessary to use this combined approach to adequately address our objectives. Furthermore, we hypothesised that AI would take a considerable time to have an impact as it would require a significant implementation period to result in measurable change. This meant that it was unrealistic to expect a measurable change in quality of working life or patient satisfaction during the period of this study; however, a qualitative analysis could illuminate whether the intervention was true to its roots in positive psychology and whether it could be implemented and help us prepare for a future evaluation. Equally, a quantitative approach remained important to enable us to test possible measures and data collection procedures for a future study.

To understand whether implementing the intervention was possible, whether staff would participate and whether the motivational ethos of AI was harnessed, we employed *qualitative* methods including ethnography and focus groups. To test the tools for data collection around staff working life, we used a *longitudinal survey* and for a patient satisfaction, we performed a *before and after* *questionnaire*. All three elements took place concurrently, supported by the same members of the research team and being inclusive of all staff at the facility who wanted to participate.

### Recruitment and consent

Written consent was sought from hospital leadership for the facility’s participation in the study. This included MHCWs being invited to the intervention sessions and agreement to the ethnography proceeding. Women were informed of the study through posters and MHCWs explaining the presence of the research team. All MHCWs on the relevant wards in each facility were invited to participate through posters, the clinical leaders (matrons and ward in-charges) informing them of the intervention session and the research team telling them about the session. Consent was not sought from individual MCHWs; they were free to attend or not and complete questionnaires for the *longitudinal survey* or not, completing the questionnaires was agreed to verbally and was taken as presumed consent. This decision was made in conjunction with the local hospitals and the ethics committees as a locally appropriate way to proceed and a way of reducing barriers to participation. All eligible MHCWs participated in at least one intervention session; although some were absent from individual sessions due to working patterns.

For the *before and after questionnaire*, the women who were currently inpatients on the postnatal ward were invited to participate. The study was explained and verbal consent obtained prior to administering the questionnaire. They formed a convenience sample with women being approached only if they were available when the research team visited.

For the *qualitative* focus groups and interviews, the staff read an information leaflet following which written consent was obtained; the focus groups and interviews were recorded and transcribed.

### Intervention

AI has 4 stages: *discovering* the best of ‘what is’; *dreaming* about the desired future; *designing* concrete proposals for a new organisational state; and implementing parts of the design in the *destiny* phase [18]. Our modification to AI was to make it into regular monthly meetings, which were held on-site monthly and lasted 1–2 h (Fig 2). The meetings were designed to rapidly breakdown barriers between staff and guide them through the four AI stages. Supplementary file 1 contains a template of the session structure for the 11 sessions. The meetings took place in staff rooms (RH and DH) and hospital meeting spaces (RH and CH). During these meetings, MHCWs sometimes made plans to work on projects between meetings; for example, they planned to come up with a way to keep the sharps bins empty and enthusiastic staff devised and implemented this mechanism between AI meetings. A Nurse-Midwife and Research Assistant facilitated meetings alongside the implementation team of MHCWs from each site.

### Data collection

The *longitudinal survey* measured changes in perceptions of staff working life using psychometrically validated scales [27–29]. The basic psychological needs scale [27] and the Quality of Working Life Scale [28] were both developed to include measures of motivational aspects of working life [27, 28]. Working-life itself was measured by elements of the Quality of Working Life Scale and also the job satisfaction and intention to leave tool [28, 29]. Together they addressed all the constructs we believed to be important for our theory of change (autonomy, competence, relatedness, job satisfaction, stress, control, home-work interface, wellbeing, conditions, engagement, intention to leave); however, none of the tools were ‘perfect’ either for our study or our setting. Two tools [27, 28] were developed and validated in high-income countries whilst the third, had been validated in African settings and used in Malawi [29] (Supplementary file 2 contains the final questionnaire and the constructs each one measured). We therefore amalgamated three scales to develop our survey, as it was not possible to develop and validate a bespoke tool for this study. We translated and back translated the three tools into the local language and piloted them with the different cadres of staff for sense and clarity. The auxiliary staff preferred to complete the tools in the local language, whereas those who were professionally qualified preferred the English version.

MHCWs completed scales pre-intervention and monthly during the 12-month study, with the exception of December as many of the staff had extended holidays so the intervention meeting was postponed until January.

The *before and after questionnaire* was used to assess patients’ satisfaction with care. The scale covered health facility, healthcare delivery and interpersonal aspects [30]. The scale chosen was originally validated for maternity services in Nepal, where, like Malawi, there is a policy for free maternity services. The 20-items covered three constructs: health facility (7 items), healthcare delivery (8 items) and interpersonal aspects (5 items). These items were answered on a four-point Likert scale ranging from completely agree to completely disagree. Two further questions were asked, firstly asking women to provide a global satisfaction rating and also whether she would deliver in this facility again [30].

The *qualitative* element aimed to identify changes occurring during the AI. The data comprised of ethnographic data, meeting minutes, facilitator reflections, end-point focus groups and individual interviews with MHCWs.

Ethnographic data was collected by the first author over the entire period of the study; this involved non-participant observation at each of the three sites, before and during the intervention period. During the observations, brief-notes were made, followed by immediate recording of detailed notes on leaving the field. The meeting minutes were taken contemporaneously by a research assistant at each meeting and used as a basis to plan the content for the next. After each meeting, the facilitators and research team (AMe, ZD, AMach) each undertook separate written reflections on how the meeting had gone.

Following the intervention, focus groups and interviews were arranged. Focus groups were used in preference to interviews, however interviews were used to bring in views (e.g. of clinical managers) who were not able to attend the focus groups. A specific interview guide was not followed, instead general questions about how AI may have worked were asked. However, all questions were in the vein of AI framing questions in a positive way for example ‘what was the best thing for you about the AI experience?’ or ‘how could AI be made better?’. During the focus groups, staff were asked to identify ‘what was important to them’ using pre-printed topic cards (e.g. teamwork) and blank cards to spark discussion.

### Sample size

For the *longitudinal survey*, all MHCWs working in the study areas were invited to participate. For the before and after questionnaire, a sample size calculation was performed to an 80% power and an alpha value of 0.05; the baseline estimates were taken from study in which this scale was developed [30]. A clinically significant change of 5 points in the 80-point scale was chosen; this required 54 women at each site pre and post-intervention. As the intervention was over the period of a year, we could not interview the same women pre-and post-intervention. After gaining consent, a research assistant verbally administered the survey using a mobile platform CommCare by Dimagi [31]. CommCare was chosen as it was developed and used in low-resource settings; it has a simple interface and records can be created online or offline.

For the *qualitative study*, MHCWs were purposefully sampled by the research team to cover all cadres at each site (clinical managers, clinical officers, nursing officers, nurse technicians, auxiliary staff).

### Data analysis

For the *longitudinal survey*, data was entered into Microsoft Excel, and STATA version 14 [32] was used to perform linear regression models to quantify the monthly change in mean scores to look at trends. We adjusted for age, gender, education, training, staff designation and length of time working at the facility. We decided to adjust for staff designation as we felt from our initial work [17] and from work in India [23] that the effects of AI may be experienced differently by different staff groups.

For the *before and after questionnaire*, due to the complexity of analysing non-paired data, a linear mixed regression model that provides flexibility analysis in correlated longitudinal data  was used to analyse the health facility, healthcare delivery and interpersonal aspects elements of the scale [33]. Descriptive statistics are presented for the overarching questions about satisfaction and using the facility again.

*Qualitative* data were as analysed using NVivo version 11 [34]. A template analysis was used [35] as a practical way to deal with a large volume of data collected over a significant time period. The initial analysis template was derived (by AMe, MLa, JH, ZD and AMa) using a modified Kirkpatrick framework [36], supported by previous qualitative work [17]. The areas covered were reaction; knowledge, skills and attitudes; behaviour change and practice changes/patient outcomes. The primary analysis was carried out by AMe, an obstetrician-in-training from the UK and PhD student, coding of some ethnographic and interview data was also carried out by MLa (a psychologist with extensive qualitative experience) and ZD (a nurse-midwife from Malawi with experience of qualitative research) coded the focus group and meeting-related data.

## Results

Table 1 shows the number and cadres of staff who attended each intervention session. The number of eligible staff reported in Table 1 represents the number of individuals invited to any session during the whole 12-month period, during the study different individuals rotated in and out of the maternity environment, meaning that at any particular time, there were fewer ‘eligible’ individuals than the total number presented here. This number fluctuated on a monthly basis.

**Table 1:** Staff attending the Appreciative Inquiry sessions

	Referral hospital		District hospital		Community hospital	
	Total	Breakdown	Total	Breakdown	Total	Breakdown
Total eligible participants*	39	3 CO	68	20 CO/MAs	47	5 CO
		20 Nurses		26 Nurses		17 Nurses
		14 Auxiliary		20 Auxiliary staff		20 Auxiliaries
		2 Clerks		2 Clerks		5 Clerks
Session 1	14	6 Nurses	23	4 CO	22	4 CO
		6 Auxiliary staff		8 Nurses		6 Nurses
		2 Clerks		8 Auxiliary staff		8 Auxiliary staff
				2 Clerks		4 Clerks
Session 2	9	4 Nurses	27	5 CO	21	3 CO
		4 Auxiliary staff		9 Nurses		5 Nurses
		1 Clerk		12 Auxiliary staff		11 Auxiliary staff
				1 Clerk		2 Clerks
Session 3	19	9 Nurses	34	6 CO/MA	22	1 CO
		8 Auxiliary staff		12 Nurses		9 Nurses
		2 Clerks		15 Auxiliary staff		10 Auxiliary staff
				1 Clerk		2 Clerks
Session 4	12	5 Nurses	26	4 CO/MA	21	3 CO
		6 Auxiliary staff		9 Nurses		5 Nurses
		1 Clerk		12 Auxiliary staff		11 Auxiliary staff
				1 Clerk		2 Clerks
Session 5	15	1 CO	36	9 CO/MA	19	3 CO
		6 Nurses		12 Nurses		5 Nurses
		6 Auxiliary staff		14 Auxiliary staff		10 Auxiliary staff
		2 Clerks		1 Clerk		1 Clerk
Session 6	14	1 CO	38	7 CO	16	2 CO
		6 Nurses		17 Nurses		3 Nurses
		5 Auxiliary staff		13 Auxiliary staff		10 Auxiliary staff
		2 Clerks		1 Clerk		1 Clerk
Session 7	16	1 CO/MA	30	4 CO/MA	20	1 CO
		6 Nurses		13 Nurses		5 Nurses
		7 Auxiliary staff		12 Auxiliary staff		12 Auxiliary staff
		2 Clerks				2 Clerks
Session 8	16	9 nurses	26	5 CO/MA	21	2 CO
		6 Auxiliary staff		9 Nurses		7 Nurses
		1 Clerk		11 Auxiliary staff		10 Auxiliary staff
				1 Clerk		2 Clerks
Session 9	19	2 CO	32	2 CO/MA	15	1 CO
		9 Nurses		15 Nurses		5 Nurses
		7 Auxiliary staff		14 Auxiliary staff		8 Auxiliary staff
		1 Clerk		1 Clerk		1 Clerk
Session 10	14	1 CO	32	4 CO/MA	18	2 CO
		6 Nurses		12 Nurses		5 Nurses
		6 Auxiliary staff		15 Auxiliary staff		10 Auxiliary staff
		1 Clerk		1 Clerk		1 Clerk
Session 11	12	4 Nurses	39	3 CO/MA	22	7 Nurses
		7 Auxiliary staff		18 Nurses		13 Auxiliary staff
		1 Clerk		17 Auxiliary staff		2 Clerks
				1 Clerk		

*CO* clinical officer, *MA* medical assistant

*Total number of eligible staff during 12-month period; however, not all were eligible at any particular time due to staffing changes and clinical rotations

In terms of implementing AI, at each of the sites 11 meetings were held (reduced from the planned 12 due to Christmas holidays). Sites progressed through the sessions at their own pace, the RH lagged behind, which from the ethnographic and reflections on the sessions, may have been because it was not embraced by the ward matron who was transferred in at the start of the study. Furthermore, at the RH the team of staff was larger and due to being based in town, they did not have hospital accommodation. Therefore, they travelled into the hospital, rather than lived on site or in the local community, making it more difficult for staff to attend on days off. Furthermore, the intervention was adopted and embraced best in the CH, where the team was smallest, and the managers were most supportive of the AI project. This commitment to the project is perhaps best illustrated by the interventions staff designed to improve their working environment or patient care. In the CH, the site observed as the one which best embraced AI, an example intervention was to improve infection prevention by taking better care with sharps bins. The team decided to not overfill bins, and to create a new rota for removal and disposal of bins which they successfully implemented, with support from the management. In the DH, they also decided to work on infection prevention and wanted to get all guardians to remove their shoes on entry to the ward. They fundraised and bought a shoe rack, and encouraged guardians to use it, which they did. In the RH, they decided to design and display posters to encourage staff to take observations more regularly and thoroughly and display them on the ward. This resulted in very little observable practice change.

We worked with the staff to arrange the meetings at times and locations suitable for them. This was largely in the afternoons after the bulk of clinical care had been carried out in the mornings. Importantly, some staff always remained on the ward to deliver care, and it was important to support this with refreshments and transport allowance being shared with the staff remaining on the wards.

Staff were willing to participate in the intervention as seen in Table 1. They maintained their attendance throughout the intervention. The clinical officers were the least likely to attend. However, from observing work patterns, they were also the least likely to be allocated solely to the wards we were working with and did not identify as strongly as being part of the core team.

To demonstrate how the data collection worked and begin to understand possible effects on staff and patients, we will report the three strands of work separately. The staff reaction elements of the study show the potential for the positive focus of AI to be maintained with this modified intervention.

### Longitudinal MHCW Survey  

One hundred forty-five MHCWs completed a survey during the study. Their only feedback about the survey itself was to shorten it. MHCW characteristics are displayed in Table 2.

**Table 2:** Characteristics of MHCWs participating in the longitudinal study

	Referral hospital (*n* = 38) *n* (%)	District hospital (*n* = 67) *n* (%)	Community hospital (*n* = 40) *n* (%)
Gender			
Female	34 (89.5)	41 (61.2)	28 (70.0)
Male	4 (10.5)	26 (38.8)	12 (30.0)
Age, mean (sd)	34.5 (7.1)	31.5 (8.6)	34.2 (6.4)
Education			
Primary	3 (8.3)	1 (1.5)	1 (2.5)
Secondary	8 (22.2)	25 (37.3)	17 (42.5)
University	25 (69.4)	41 (61.2)	22 (55.0)
Training			
Certificate	4 (10.8)	9 (13.4)	4 (10.0)
Degree	5 (13.5)	8 (11.9)	2 (5.0)
Diploma	13 (35.1)	30 (44.8)	16 (40.0)
n/a	15 (40.5)	20 (29.9)	18 (45.0)
Designation			
Auxiliary	16 (42.1)	21 (31.3)	18 (45.0)
Clinical	3 (7.9)	19 (28.4)	5 (12.5)
Nursing	19 (50.0)	27 (40.3)	17 (42.5)
Length of time in months working at facility, mean, sd	102.9 (69.4)	54.2 (65.5)	100.7 (70.6)

Table 3 shows the results for each of the working life scales. Unadjusted and adjusted estimates did not differ substantially; therefore, adjusted estimates are presented.

**Table 3:** Summary of the average increase in the mean score per month (b) for each of the three working life scales in MCHWs, with a breakdown by site and cadre of staff

	Overall	Auxiliary staff^**+**^	Clinical staff ^**++**^	Nursing staff
	b* (95% CI)	b* (95% CI)	b* (95% CI)	b* (95% CI)
**Referral hospital**				
Basic psychological	0.00 (− 0.03, 0.03)	− **0.04** **(**− **0.08,** − **0.01 )**	0.12 (− 0.11, 0.35)	0.019 (− 0.02, 0.05)
Job satisfaction	0.01 (− 0.02, 0.04)	− 0.02 (− 0.06, 0.03)	− 0.17 (− 0.47, 0.13)	0.034 (− 0.02, 0.08)
Work-related quality of life	0.02 (− 0.01, 0.05)	− 0.01 (− 0.05, 0.03)	0.08 (− 0.17, 0.3)	**0.05** **(0.01, 0.08)**
**District hospital**				
Basic psychological	− 0.01 (− 0.02, 0.00)	− **0.03** **(**− **0.05,** − **0.00 )**	0.01 (− 0.04, 0.05)	0 (− 0.02, 0.02)
Job satisfaction	0.01 (− 0.01, 0.00)	0.00 (− 0.02, 0.02)	0.01 (− 0.04, 0.06)	0.01 (− 0.02, 0.03)
Work-related quality of life	0.01 (− 0.00, 0.02)	0.01 (− 0.01, 0.03)	− 0.02 (− 0.06, 0.02)	0.02 (− 0.00, 0.04)
**Community hospital**				
Basic psychological	0.02 (− 0.00, 0.04)	**0.03** **(0.00, 0.06)**	0.02 (− 0.04, 0.08 )	− 0.01 (− 0.04, 0.03)
Job satisfaction	0 (− 0.02, 0.02)	0.01 (− 0.02, 0.04)	− 0.02 (− 0.08, 0.05 )	− 0.02 (− 0.05, 0.02)
Work-related quality of life	**0.02** **(0.00, 0.04)**	**0.03** **(0.01, 0.06)**	0.02 (-0.03, 0.07)	0.01 (− 0.02, 0.04)
**Overall**				
Basic psychological	0 (− 0.1, 0.01)	− 0.01 (− 0.02, 0.01)	0.02 (− 0.02, 0.06)	0.00 (− 0.01, 0.02)
Job satisfaction	0.00 (− 0.01, 0.02)	0.00 (-0.01, 0.02)	− 0.00 (− 0.04, 0.04)	0.01 (− 0.02, 0.03)
Work-related quality of life	**0.02** **(0.01, 0.02)**	**0.02** **(0.00, 0.03)**	− 0.01 (− 0.04, 0.03)	**0.02** **(0.00, 0.04)**

Adjusted results reported as minimal difference between un-adjusted and adjusted

*b refers to the average increase in the mean score per month

^+^Auxiliary staff are those who are non-professionally qualified and include hospital attendants, patient Attendants, nursing auxiliaries, clerks and messengers

^++^ Clinical staff are Clinical Officers and Medical Assistants.

When combining the results across all three sites and all cadres of staff, there was no evidence of significant change in the basic psychological needs scale or job satisfaction scales. However, the work-related quality of life scale showed improvement both overall and within the specific groups of auxiliary and nursing staff.

When considering the results by hospital, apart from the basic psychological needs scale in the DH, there was an increase in the scores. However, there was only strong evidence in favour of the intervention working in the CH in the work-related quality of life scale.

When considering the results by site and cadres of staff, the auxiliary staff at the RH experienced a decrease in satisfaction with work; there was only strong evidence for this in the basic psychological needs aspect of the survey. This contrasts with the auxiliary staff at the CH where there was strong evidence of an improvement in working life in the basic psychological needs and work-related quality of life scales. In the DH, there was evidence of a decrease in the basic psychological needs scale but this was not the case in the other scales.

Nurses at the RH displayed strong evidence of increases in the work-related quality of life scale only. The CH nurses showed weak evidence of a downward trend in both the basic psychological needs survey and the job satisfaction tool. The clinical officers and medical assistants showed mixed results in all sites.

### Before and After Questionniare of Patient satisfaction

All women who were approached by the study team agreed to participate. The use of the CommCare app received positive feedback from the research team. The characteristics of participants are shown in Table 4, which were comparable before and after the intervention within each site.

**Table 4:** Characteristics of women participating in the study

	Referral hospital		District hospital		Community hospital	
	Before *n* = 56	After *n* = 60	Before *n* = 54	After *n* = 62	Before *n* = 52	After *n* = 69
Mean age (SD)	25.7 (6.3)	26.5 (6.5)	25.4 (5.5)	25.2 (7.9)	23.5 (5.3)	24.1 (5.8)
Mean travel time min (SD)	67.5 (47.5)	48.3 (26.4)	110 (39.5)	76.0 (47.2)	94.3 (51.4)	67.9 (39.1)
Primiparous *n* (%)	23/56 (41%)	19/60 (32%)	20/54 (37%)	25/62 (40%)	19/52 (37%)	31/69 (45%)
Educational level Primary or less *n* (%)	34/56 (61%)	35/69 (58%)	44/54 (81%)	49/62 (79%)	45/52 (87%)	57/69 (83%)

Women’s satisfaction with care is shown in Table 5. It rose convincingly in the DH and CH but less so in RH. In the RH, the ‘health facility’ element was lower post-intervention. All other components increased across sites. The adjusted comparisons (age, number of children, time taken to travel and educational level) revealed larger differences than unadjusted.

**Table 5:** A before and after comparison of change in patient satisfaction scores and global satisfaction scores

	**Change in satisfaction scores before and after AI**					
	Referral hospital		District hospital		Community hospital	
	Unadjusted	Adjusted	Unadjusted	Adjusted	Unadjusted	Adjusted
Health facility (95% CI)	-0.65 (-1.62 to 0.31)	0.05 (-1.02 to 1.12)	2.65 (1.68 to 3.61)	2.96 (1.79 to 4.13)	3.78 (2.82 to 4.73)	3.92 (2.84 to 5.00)
Healthcare delivery (95% CI)	1.60 (0.67to 2.52)	1.92 (0.82 to 3.01)	3.42 (2.46 to 4.38)	4.02 (2.80 to 5.24)	4.06 (3.14 to 4.97)	4.28 (3.18 to 5.37)
Interpersonal aspects (95% CI)	1.74 (0.72 to 2.76)	2.35 (1.17 to 3.52)	2.55 (1.53 to 3.57)	3.27 (2.00 to 4.54)	4.98 (3.98 to 5.99)	4.78 (3.60 to 5.95)
Overall (95% CI)	2.57 (0.30 to 4.83)	4.41 (1.89 to 6.95)	8.53 (6.17 to 10.88)	10.22 (7.38 to 13.07)	12.82 (10.56 to 15.08)	13.02 (10.48 to 15.57)
	**Were women completely satisfied with care?**					
	Referral hospital		District hospital		Community hospital	
	Before *n* = 56	After *n* = 60	Before *n* = 54	After *n* = 62	Before *n* = 51	After *n* = 69
Completely agree	23 (41.1%)	42 (70.0%)	9 (16.7%)	45 (72.9%)	6 (11.5%)	59 (85.5%)
Agree	23 (41.1%)	9 (15.0%)	42 (77.8%)	9 (14.5%)	33 (63.5%)	5 (7.3%)
Disagree	4 (7.1%)	7 (11.7%)	2 (3.7%)	5 (8.1%)	6 (11.5%)	4 (5.8%)
Completely disagree	6 (10.7%)	2 (3.3%)	1 (1.85%)	3 (4.8%)	6 (11.5%)	1 (1.5%)
	**Women would use the facility for a future delivery**					
	Referral hospital		District hospital		Community hospital	
	Before *n* = 56	After *n* = 60	Before *n* = 54	After *n* = 62	Before *n* = 51	After *n* = 69
Yes	39 (69.6%)	50 (83.3%)	50 (92.6%)	58 (93.6%)	41 (78.9%)	66 (95.7%)
No	11 (19.6%)	8 (13.3%)	3 (5.6%)	2 (3.23%)	9 (17.3%)	2 (2.9%)
Undecided	6 (10.7%)	2 (3.3%)	1 (1.9%)	2 (3.23%)	2 (3.9%)	1 (1.45%)

The overall satisfaction scores show that there is a move towards being more satisfied after the intervention. However, in terms of future delivery, there is no clear  change in the number who would choose to deliver at that facility again, except in the CH.

### Qualitative data

Fifteen individual interviews and 8 focus groups supplemented ethnographic data and meeting reports. During the analysis, the template was developed as themes emerged.

A site-specific summary is presented in Supplementary files 3, 4 and 5. Many changes were remarkably similar across sites, so a general summary is reported below.

### Reaction

Staff enjoyed AI meetings as they were able to voice their opinions and were treated respectfully. During meetings, we observed that all cadres of staff actively participated and were enthusiastic to arrange the next meeting. They felt that AI meetings built relationships*:* ‘I am happy and I would want it to be sustained because we are in very good relationship with our supervisors and we feel motivated to come to work’ hospital attendant from DH. Furthermore, they believed that AI meetings catalysed change: ‘we have seen things changing, positive things happening’ Matron CH.

Removing the focus on the negatives was motivating. ‘It’s like it motivates when you have positive mind or positive things. They bring praise to you. It encourages everybody... previously…people have been saying negative things...It demotivates…people.’ RH, Ward Clerk

### Change in knowledge, skills or attitude

MHCWs understood team members capabilities and listened to each other. A DH nurse felt ‘we were coming with suggestions as a group not as an individual…So it really helped us strengthen the teamwork because we were doing things together...’

Improved communication and contribution was valued. MHCWs reported that their communication had improved, a nurse from the RH shared ‘I have learnt how to interact with people…and…our patients.’

MHCWs wanted to gain new knowledge and skills. At the RH and CH, non-trained staff were taught to perform observations. Staff increased awareness using posters highlighting handwashing, visiting times, waste segregation and observations.

Staff felt that working life improved with teamwork. The CH ward clerk explained ‘I have seen that now my work is enjoyable… because of this AI, we have got team spirit’.

A hospital attendant from the RH described ‘our work to be enjoyable now. In the past, we would stay away from work because we had quarrelled with our colleague or because of unpleasant work environment. But now, staying away from work for no apparent reason is unthinkable, we are anxious to go to work because our relationship is good.’

### Behaviour change

Relationships becoming more respectful and more equal interactions  emerged clearly: ‘As juniors, we were taunted most of the time, or let me say that we were discriminated against, but because of this project, we feel that now we are able to relate to each other and are united in our work.’ RH, Hospital Attendant.

The team changed their working: ‘Previously, I don’t think that we discussed anything…We were just working individually, but because of the AI, we are able to meet and then discuss.’ Clinical Officer, DH

Staff felt they were working better together, supporting each other to achieve goals. A CH nurse explained their use of ‘AI’ as a slogan ‘to remove embarrassment to our friends; because sometimes you could just say, “You have not washed your hands and now you are touching the patient!” but if just say, “AI” That friend of yours knows that, “I have made a mistake somewhere, maybe I have not washed my hands” So she checks herself, “Where have I gone wrong!” So with the slogan of AI it has really moved the embarrassment of our staff.’

The improved teamworking was also observed in the ethnographic data. One example being in the DH where auxiliary nurses from both wards began to work together to move patients between the theatres/labour ward and the postnatal ward. Previously, this had not been a shared task. Furthermore, in the meeting reports and reflections as the intervention progressed there was increasing interaction from all cadres of staff. This was particularly noticeable in the less well-qualified members of staff. They became increasingly engaged in the process as the AI meetings continued.

### Changes in practice or patient outcomes

These changes were hospital specific. As AI continued, staff were less likely to take time off work. In the CH, the Deputy-in-charge believed ‘the rate of sick leaves has actually gone down so it’s a better achievement.’

Staff developed systems; staff emptied the sharps bins and monitored the incidences of the bins becoming overfull (CH); teams made rota’s for health talks for traffic control (DH); and handwashing (CH).

Staff wanted to empower patients. They put posters on the walls, arranged health talks and communicated individually. In the RH, they informed patients about ward systems meaning that ‘Patient[s] are around, when we explain [to] them on[at] this time you should be available for drug [rounds at] this time for vital sign[s] patient’s are always available’ Nurse, RH.

Staff felt clinical care and outcomes had improved. At the DH, a nurse explained how ‘neonatal sepsis…and puerperal sepsis also has been reduced since patients and guardians are able to follow IP[Infection Prevention] protocols’. However, when observing practice, it was not clear that clinical care and outcomes had improved. Changes in practice were observed, for example in the DH the guardians had a designated place to leave their shoes, and staff worked together to push patients between wards. However, there was no evidence of improved outcomes.

## Discussion

We have shown that it is possible to implement AI in Malawi, and that although we do not have strong evidence, as this was a non-randomised feasibility study, we believe that we have identified that AI has the potential to effect working life and patient satisfaction. We have also seen that it was welcomed both by the District Health Officers who provided permission and advice for study sites, as well as all cadres of staff. Alongside this, we have explored both patient and staff experience measures to consider in a future trial.

The theory outlined in Fig. 1 is a useful framework for considering AI’s possible impact. This study aimed to see if AI could address motivational factors, and ultimately that this could lead to improved patient care. Whilst this study was not long enough to mediate improved clinical outcomes or MHCW retention, it has shown that it may be possible to use AI to effect change both in motivation and outcomes. This suggests that AI may mediate behaviour change. One way this effect can be understood is to draw upon a well-known behavioural change model (COM-B) which outlines the elements required to effect behaviour change: capabilities, opportunity and motivation [37]. When considering AI in this way, the plausibility of it changing outcomes emerges. Undertaking an intervention which has been approved by the management provides staff with opportunity through creating the time and the ‘permission’ to participate. By participating in the intervention, they gain the capability to make changes. AI specifically attempts to motivate people, and thus combined there is the possibility of moving towards changing behaviour.

Working lives were impacted through the important alterations in inter-personal interactions that it fosters. MHCWs enjoyed participating in AI and all eligible MHCWs joined the meetings where possible. The qualitative component showed that AI facilitated changes in attitudes and behaviour which staff valued and believed would be lasting. They discussed altered interactions and new ways of doing things leading to systematic changes and alterations in patients’ care.

Strikingly, staff unanimously reported relationships had changed. This seemed to underpin the other changes. The lower cadres felt valued, and seniors realised everyone could contribute. This is important as teams exhibiting less hierarchy, deliver better patient outcomes [38], in part because in hierarchies staff are not empowered to speak up [39]. Interpersonal relationships seem to be an important factor in the psychological well-being of staff [14], and the fact that AI could foster progress to improving these relationships is important for motivation as a whole.

The longitudinal survey showed positive changes in working-life scale in the study overall, and in particular in the nurses and auxiliary staff. When considering individual sites and staff groups, there were mixed results. Interestingly, there was a reduction across all scales (although only significant in the basic psychological needs scale) for the auxiliary staff in the RH and in the basic psychological needs scale in the DH, but an increase across all scales in the CH. One explanation may be due to how the intervention was adopted in each site, with it being embraced best in the CH and least in the RH. The auxiliary staff enthusiastically attended and participated in AI; however, sometimes the planned actions were not embraced by all members of the team (e.g. nurses and clinical officers), and the auxiliary staff may have found this frustrating. This may have been because the intervention had the largest impact on their working lives as they were previously least empowered. This idea is in keeping with other studies of AI [22, 23], where the most important changes were described as interpersonal changes which had the largest effect on the most junior staff.

In terms of effects on clinical officers, there were no significant changes, and there were no clear trends in any site or scale. The results for the nurses were again mixed, but with significant improvements in the work-related quality of life scale in the RH and DH. This may have been due to the fact that the clinical officers worked across the whole hospital whereas the nurses were embedded in the wards and the initiatives that were implemented were often focussed at the ward level.

The total scores across all three scales are relatively low, suggesting low satisfaction with working life generally. Which is in keeping with a recent study which found low psychological well-being of healthcare workers in Malawi [14]. Because of these low scores, the overall changes in the mean scores occurred within a very small window and therefore determination of a change during or following the intervention is difficult.

Patient satisfaction improved following AI when considering the broad areas of the healthcare facility, healthcare delivery and interpersonal aspects. Interestingly, the CH performs best across all areas and RH the worst. There may be several contributory factors. One possibility is that the RH is has a large number of deliveries so is busy resulting in it being harder to provide high-quality care; furthermore, it is in town and women who attended it were more highly educated; this may mean that women have a different set of expectations. It has been suggested that a lack of knowledge results in low expectations of care in Malawi [40], and therefore this higher level of education compared to the other sites could go some way towards explaining this. However, so could the fact that the RH was the busiest hospital with the most patients. Due to the study design, it is not possible to definitively attribute this to AI.

The health facility components were stable in the RH but increased in CH and DH. This is remarkable because the physical alterations during the study were minimal. One possible explanation is that any increase in the health facility scores was moderated by the increase in the interpersonal skills of MHCWs. This resonates with the other AI studies from resource poor settings, for example, in India [23], improvements in patient’s satisfaction with staff’s actions was documented.

It is interesting to consider the results of the working life and patient satisfaction scales with the perspective of the successfulness of the implementation of the interventions. The study team (and the altered timetable) suggest that the CH adopted the intervention most enthusiastically and the RH was most challenging site for implementation. This makes the fact that the results for any changes seem most marked in the CH and least in the RH particularly interesting from the perspective of considering the effects of implementation on outcomes. Considering a clear framework for assessment of implementation fidelity is likely to be important to support the implementation of AI in a wider study. An on-going assessment of key implementation elements would allow early identification and intervention to improve the fidelity of the implementation of AI and thus the outcomes.

### Strengths and limitations

We believe that whilst the mixed methods approach may have placed an additional burden on the participating healthcare workers, in so far as they had to complete the questionnaires and were also invited to attend focus groups/interviews, it did not place any significant limitations on any element of the study. Mixed methods have enabled us to deepen our understanding of elements which are less well reflected in a survey as they are more complicated relationship and organisational factors.

The changes in the scales could have occurred by chance or due to other system changes and our study was not powered to provide definitive evidence of effect. Having said this, it is encouraging to note that whilst across all sites the changes implemented were different, the general messages were remarkably consistent. The staff had better relationships, changed how they worked and interacted better with colleagues and patients and patient satisfaction improved.

A limitation of the study came with the scales; the basic psychological needs and work-related quality of life scales were not validated in LMIC settings, nor was it possible to do so given the resource limitations. Furthermore, the combined questionnaire was lengthy. In terms of the patient study, we did not collect the qualitative data from the women which may have provided deeper insights into the differences between sites.

A key limitation was that we did not measure implementation fidelity. In a future study, it will be important to develop a tool to record features such as implementation factors (e.g. number, length, attendance and content of meetings); organisational factors (for example managerial support and availability of time to participate); and interpersonal factors (for example, whether all team members contribute to the meetings and various activities between meetings).

A further element to evaluate in a future study would be the capabilities and opportunity elements of the behaviour change model (COM-B) [37]. This would enable a deeper understanding and identification of the key elements required to result in successful behaviour change, and thus help develop the vital elements for a wider roll-out.

## Conclusion

AI is feasible to implement in government facilities in Malawi and has the potential to affect the MHCWs working life and improve the way patient’s experience care. To understand whether this is worth significant investment, large-scale studies of AI in maternity settings in Malawi and other resource poor settings should be undertaken to determine its effectiveness.

## Supplementary Information

**Additional file 1.** Session outlines for the 11 session Appreciative Inquiry Intervention.

**Additional file 2.** Staff longitudinal survey questionnaire and constructs addressed  within it.

**Additional file 3.** Qualitative summary from the Referral Hospital.

**Additional file 4.** Qualitiative summary from the District Hospital.

**Additional file 5.** Qualitative summary from  the Community Hospital.

## Data Availability

The datasets used and/or analysed during the current study are available from the corresponding author on reasonable request.
