# Supplementary material for: Assessing the impact of a motivational intervention to improve the working lives of maternity healthcare workers: a quantitative and qualitative evaluation of a feasibility study in Malawi
Source: Pilot Feasibility Stud. 2021 Jan 29;7:34. doi: 10.1186/s40814-021-00774-7 (PMC7844964; doi:10.1186/s40814-021-00774-7)
Supplement: Supplementary file 1 — Additional file 1. Session outlines for the 11 session Appreciative Inquiry Intervention. [file 40814_2021_774_MOESM1_ESM.docx]

**Session outlines for the 11 session Appreciative Inquiry Intervention**

| **Session 1:**  Introductions to AI team and project  Introduction to AI:  Appreciative Q 1: in pairs/groups (5 mins- feedback 5 mins)  *What has been your single best moment whilst working on the high risk postnatal ward and why?*  Appreciative Q 2: in different pairs/groups (5 mins feedback 5 mins)  *What is it that you value most about yourself?*  *What is it that you value most about your work?*  *What is it that you value most about the postnatal ward?*  Appreciative Q3: in different pairs/groups (5 mins feedback 5 mins)  *What is the core/important thing that gives the postnatal ward life?*  Appreciative Q 4: in different pairs/groups (5 mins feedback 5 mins).  *If you had three wishes for the postnatal ward what would they be?*  Create list of possible topics  Vote for the top choice topic (post-its or voting with feet)  Commit to interviews and understand collection and distribution system (5mins) |
| --- |

| Session 2:   - Quick reminder of names and project (2 mins) - Brief feedback from the group about the discovery activity – invite people to share their favourite stories from the first round. Approximately 3. (10 mins) - High point analysis – thematic feedback from the first round (5 mins) - Energiser (5 mins) - Now for the task for the day - in small groups – write on sheets and one person present back after 15 mins - you will have 5 mins to present back.   - You wake up tomorrow and your ward is as you wished and dreamed it would be:     - What is happening     - How is it different     - What are you doing that makes a difference. |
| --- |
| **Session 3:**   - Introduction of the team (3 mins) - Sum up of AI (5 mins) - Recap of Discover and Dream phases (5 mins) - Identify the recurring themes from the ‘Dream Phase’ - Then you will have 10 minutes to discuss these as a group - create up to 5 provocative propositions that you agree on as a group - present back their provocative propositions. |

| **Session 4**   - Recap of the AI so far: (10 mins)   - Now we’re going to remind you of what we’ve done using the SOAR process: Strengths/opportunities/Aspirations/Results.   - Strengths to build on: xxxx   - Opportunities: xxxx   - Aspirations: provocative Propositions xxxxx - This week we’re going to work on the destiny phase and also remember that we need to make sure that we see results so you know just how much you’re achieving as a team!   To start with just a little about this destiny phase:  This is your opportunity to declare what actions you’d like to take and then get support from the other members in your organisation.  This is the start of an appreciative learning culture, this is where you take the positives that we have been discussing and harness them and build upon them.   - Todays task:   We invite you to look at the provocative propositions pinned around the room and choose if there is one you would like to work on. You need to care and be passionate about it.  This is the opportunity to develop your action plans  When you do this you need to rember:   - focus on strengths - be passionate - ensure that there is ongoing collaboration - Remember that you need to be able to measure the change so that you can see it happening!   Here’s how it will happen:   1. Talk about the provocative proposition and make sure that you agree on the working of the proposition 2. Spend 15 mins brainstorming things that you can do as a team to build on the successes here at Dowa and towards the provocative proposition. 3. You can spend 5 mins choosing the thing that you most care about as a group ( they could always split into two if that was better). 4. For 15 mins you can plan what you will do towards your idea. 5. For 10 mins you can plan how you as a group will monitor this change   Feedback each group will have just 2 mins to present their idea |
| --- |

| **Session 5**  **Introduction to AI: 5 mins**  Recap of where we’re up to at site x – with feedback from the team members who are present (10 mins):   - We chose the topic of : - We interviewed each other and identified the following high points in the discovery phase: - we then dreamed about the xxx at its best: - we made the following provocative propositions:xxx   Current action plans: report from the team.  Energiser  Consider our strengths as individuals: card game (using ‘at our best’ cards)  In groups of 3-5 – discuss for 5 mins how the xxxx hospital/ward would be at its best  Provocative propositions: Do we agree with them? Is the focus of them on what’s most important to you?  How are the action plans going, what can the team do to support success? |
| --- |

| **Session 6**  Recap of last meeting:  Review of each action plan  Work together in groups to build on action plan  Strengths cards game – what is the strength of your partner  Then action plan final decision and team commitment |
| --- |

| **Session 7**  Recap of last meeting’s decisions (10 mins)  Report of what’s being going on between sessions  Celebrate successes – make a poster of the successes since the last session.  Review and adapt action plans  Are we happy concentrating on current action plans or do we want to do a new area too?  Can use the wand game….to consider old provocative propisions again or consider a new one:  Get into groups of 5 with a wand. Whoever has hold of the wand shares with their group their dream for one of the current priority topics (5 mins) |
| --- |

| **Session 8 – 11**  Every session focus on recapping previous decisions  Assessing progress making sure to focus on successes  Strengthen action plans and try new things  Is there anything we need to change to make the successes even stronger? |
| --- |
