# Supplementary material for: Assessing the impact of a motivational intervention to improve the working lives of maternity healthcare workers: a quantitative and qualitative evaluation of a feasibility study in Malawi
Source: Pilot Feasibility Stud. 2021 Jan 29;7:34. doi: 10.1186/s40814-021-00774-7 (PMC7844964; doi:10.1186/s40814-021-00774-7)
Supplement: Supplementary file 2 — Additional file 2. Staff longitudinal survey questionnaire and constructs addressed within it. [file 40814_2021_774_MOESM2_ESM.docx]

**Staff longitudinal survey questionnaire and constructs within it**

Constructs:

**Questionnaire to understand working life experience of healthcare workers**

| **Firstly we would like to understand a little more about you and your role. Please fill in the blank boxes or circle the appropriate answer.** | |
| --- | --- |
| How old are you? |  |
| What is your gender? | Male / Female |
| Highest Educational qualification |  |
| Professional training | None / Degree in clinical medicine / Certificate in clinical medicine / Nursing degree / Diploma or certificate in nursing / Diploma or certificate in environmental health /  don’t know or can’t say / Other (please specify) …………………………………. |
| Designation | Medical assistant / Clinical officer / Doctor / Nurse / Health assistant / HSA /  Ancillary Staff / don’t know or can’t say / Other (please specify)………………………… |
| How long have you worked in this role? |  |

Please read the questions and answer with your first thoughts, you do not need to take a long time to think about the answers. This is not a test, it will just help us to understand the factors affecting your experience at work. Please place a tick (✔) in the box you think most represents how you feel. Remember your boss will never know how you personally responded to this questionnaire.

| The following questions concern your job in the last year (or the total time in this job if less than a year). Please indicate how true each of the following statements is for you given your experiences on this job. | | Not at  all true | | Somewhat true | | | Very true | |
| --- | --- | --- | --- | --- | --- | --- | --- | --- |
|  |  | 1 | 2 | 3 | 4 | 5 | 6 | 7 |
| 1 | I feel like I can make a lot of inputs to deciding how my job gets done. |  |  |  |  |  |  |  |
| 2 | I really like the people I work with |  |  |  |  |  |  |  |
| 3 | I do not feel very competent when I am at work |  |  |  |  |  |  |  |
| 4 | People at work tell me I am good at what I do |  |  |  |  |  |  |  |
| 5 | I feel pressured at work |  |  |  |  |  |  |  |
| 6 | I get along with people at work |  |  |  |  |  |  |  |
| 7 | I pretty much keep to myself when I am at work |  |  |  |  |  |  |  |
| 8 | I am free to express my ideas and opinions on the job |  |  |  |  |  |  |  |
| 9 | I consider the people I work with to be my friends |  |  |  |  |  |  |  |
| 10 | I have been able to learn interesting new skills on my job |  |  |  |  |  |  |  |
| 11 | When I am at work, I have to do what I am told |  |  |  |  |  |  |  |
| 12 | Most days I feel a sense of accomplishment from working |  |  |  |  |  |  |  |
| 13 | My feelings are taken into consideration at work |  |  |  |  |  |  |  |
| 14 | On my job I do not get much of a chance to show how capable I am |  |  |  |  |  |  |  |
| 15 | People at work care about me |  |  |  |  |  |  |  |
| 16 | There are not many people at work that I am close to. |  |  |  |  |  |  |  |
| 17 | I feel like I can pretty much be myself at work |  |  |  |  |  |  |  |
| 18 | The people I work with do not seem to like me much |  |  |  |  |  |  |  |
| 19 | When I am working I often do not feel very capable |  |  |  |  |  |  |  |
| 20 | There is not much opportunity for me to decide for myself how to go about my work |  |  |  |  |  |  |  |
| 21 | People at work are pretty friendly towards me. |  |  |  |  |  |  |  |

|  | To what extent do you agree with the following?  Please tick (✔) the appropriate box. | Strongly Disagree | Disagree | Neutral | Agree | Strongly agree |
| --- | --- | --- | --- | --- | --- | --- |
| 1 | In general, I am satisfied with this job |  |  |  |  |  |
| 2 | I feel that I am able to use my abilities to their full potential |  |  |  |  |  |
| 3 | I have a variety of duties, tasks and activities in my job |  |  |  |  |  |
| 4 | I find that my opinions are respected at work |  |  |  |  |  |
| 5 | I am satisfied with the recognition I get for the work that I do |  |  |  |  |  |
| 6 | I am satisfied with the personal relationship between my manager and myself |  |  |  |  |  |
| 7 | I am satisfied with the way my manager handles staff |  |  |  |  |  |
| 8 | I feel that my job conditions allow me to perform at high levels |  |  |  |  |  |
| 9 | I am satisfied with the availability of drugs and equipment |  |  |  |  |  |
| 10 | I am satisfied with the education/training opportunities that I get |  |  |  |  |  |
| Please note this questionnaire continues on the next page…. | | | | | | |
|  | **To what extent do you agree with the following?**  Please tick (✔) the appropriate box. | **Strongly Disagree** | **Disagree** | **Neutral** | **Agree** | **Strongly agree** |
| 11 | I am actively seeking other employment |  |  |  |  |  |
| 12 | I have a clear set of goals and aims to enable me to do my job |  |  |  |  |  |
| 13 | I feel able to voice opinions and influence changes in my area of work |  |  |  |  |  |
| 14 | I have the opportunities to use my abilities at work |  |  |  |  |  |
| 15 | I feel well at the moment |  |  |  |  |  |
| 16 | My employer provides adequate facilities and flexibility for me to fit work in around my family life |  |  |  |  |  |
| 17 | My current working hours/ patterns suit my personal circumstances |  |  |  |  |  |
| 18 | I often feel under pressure at work |  |  |  |  |  |
| 19 | When I have done a good job it is acknowledged by my line manager |  |  |  |  |  |
| 20 | Recently, I have been feeling unhappy and depressed |  |  |  |  |  |
| 21 | I am satisfied with my life |  |  |  |  |  |
| 22 | I am encouraged to develop new skills |  |  |  |  |  |
| 23 | I am involved in decisions that affect me in my own area of work |  |  |  |  |  |
| 24 | My employer provides me with what I need to do my job effectively |  |  |  |  |  |
| 25 | My line manager actively promotes flexible hours/patterns |  |  |  |  |  |
| 26 | In most ways my life is close to ideal |  |  |  |  |  |
| 27 | I work in a safe environment |  |  |  |  |  |
| 28 | Generally things work out well for me |  |  |  |  |  |
| 29 | I am satisfied with the career opportunities available for me here |  |  |  |  |  |
| 30 | I often feel excessive levels of stress at work |  |  |  |  |  |
| 31 | I am satisfied with the training I receive in order to perform my present job |  |  |  |  |  |
| 32 | Recently, I have been feeling reasonably happy all things considered |  |  |  |  |  |
| 33 | The working conditions are satisfactory |  |  |  |  |  |
| 34 | I am involved in decisions that directly affect members of the public |  |  |  |  |  |
| 35 | I have unachievable deadlines |  |  |  |  |  |
| 36 | My work is as interesting and varied as I would want it to be |  |  |  |  |  |
| 37 | I am able to achieve a healthy balance between my work and home life |  |  |  |  |  |
| 38 | I feel motivated to do my best in my current job |  |  |  |  |  |
| 39 | The organisation communicates well with its employees |  |  |  |  |  |
| 40 | I am proud to tell others that I am part of this organisation |  |  |  |  |  |
| 41 | I would recommend this organisation as a good one to work for |  |  |  |  |  |
| 42 | I get a sense of achievement from doing my job |  |  |  |  |  |
| 43 | I am pressured to work long hours |  |  |  |  |  |
| 44 | I have unrealistic time pressures |  |  |  |  |  |
| 45 | I have sufficient opportunities to question managers about change at work |  |  |  |  |  |
| 46 | I am happy with the physical environment where I usually work |  |  |  |  |  |
| 47 | I am satisfied with the overall quality of my working life |  |  |  |  |  |

**If there is anything else that important to your every day working life that you would like to tell us more about please write about it here………………………………………………………………………………………………………………………………………………………………………………………………………………………………….…………………………………………………………………………………………………………………………………….**

**…………………………………………………………………………………………………………………………………….**

Thank you for completing this questionnaire. We will feed back the results to you during the action cycle meetings.
