# Supplementary material for: Assessing the impact of a motivational intervention to improve the working lives of maternity healthcare workers: a quantitative and qualitative evaluation of a feasibility study in Malawi
Source: Pilot Feasibility Stud. 2021 Jan 29;7:34. doi: 10.1186/s40814-021-00774-7 (PMC7844964; doi:10.1186/s40814-021-00774-7)
Supplement: Supplementary file 3 — Additional file 3. Qualitative summary from the Referral Hospital. [file 40814_2021_774_MOESM3_ESM.docx]

| Kirkpatrick area & sub themes | Examples in the Referral Hospital |
| --- | --- |
| Reaction | |
| Exciting/fun | Enthusiasm in meeting |
| Positive Experience | Motivating experience, |
| Should Continue | Want meetings to continue to sustain change |
| Useful | AI is helpful to the ward and means they don’t get discouraged |
| Knowledge/Skills/Attitudes |  |
| Appreciating each other | Appreciating the work of all cadres |
| Better understanding of each other | Meetings mean everyone can provide views and can all work on the same things |
| *Happier/Easier work* | Less discrimination of juniors, feel happier so work harder, HA’s doing tasks only nurses used to do |
| *Improved non-technical skills* | Improved communication between staff groups |
| Improved Knowledge | HA’s taught to perform observations |
| Improved resilience | More willing to help each other when there are problems or when other staff are busy. Willing to find solutions e.g. using mobile phones as watches |
| Lobbying for change | Lobbying for resources e.g. BP machines |
| *Raising awareness to improve care* | Posters to remind staff of observations and politeness |
| *Empowered, pride/respect in work* | HA’s taking responsibility for patients as more valued, staff in general taking responsibility for tasks, feel empowered, |
| Behaviour Change |  |
| Altered interactions with staff/patients | Communal lunch re-instated, HA’s communicate with nurses when there is a problem with observations/patients, feel they communicate better with patients and other members of staff, welcome the patients to the ward, better handovers between staff e.g. of where the resources are |
| Altered supervision/feedback methods | More coordination between supervisors and junior staff, new approach to supervision, Juniors feel supervisors are more respectful |
| *Individual altering behaviour* | Monitoring respirations which they were not doing before, Leading by example, new way for managing staff |
| *Monitoring change* | Designed and carried out patient satisfaction survey, re-introduced suggestions box, monitoring observation taking with audit meaning that people taking more responsibility |
| New forums to discuss ideas | Time to discuss things at communal lunch |
| Team alter way of working | Task allocation for admissions and observations, plans to work with non-ward staff to protect the new linen and improve respectful care, team to contribute towards lunch equipment, more regular monitoring of patient observations, HA’s take observations, all staff working together better e.g. moving patients to and from theatre as a team, nurses trust HA’s more leaving them with keys and responsibility, taking better care of resources, ward known as best stocked ward with no stealing due to trust in team. |
| Practice Changes/Patient Outcomes |  |
| *Patients changing behaviour* | Being more available for observations |
| Development of protocols/guidelines/systems | New system of nurses/HA’s working in teams |
| Improved patient satisfaction | Feeling that patients more satisfied as good feedback from questionnaires and suggestions box |
| Improved retention/recruitment/sickness | Staff report they are less likely to call in sick as they are happier at work |
| *Information shared with patients/relatives* | Share information with patients about how the ward works |
