# Supplementary material for: Assessing the impact of a motivational intervention to improve the working lives of maternity healthcare workers: a quantitative and qualitative evaluation of a feasibility study in Malawi
Source: Pilot Feasibility Stud. 2021 Jan 29;7:34. doi: 10.1186/s40814-021-00774-7 (PMC7844964; doi:10.1186/s40814-021-00774-7)
Supplement: Supplementary file 4 — Additional file 4. Qualitiative summary from the District Hospital. [file 40814_2021_774_MOESM4_ESM.docx]

| Kirkpatrick area & sub themes | Examples in the District Hospital |
| --- | --- |
| Reaction | |
| Exciting/fun | Meetings enjoyable and high energy |
| Positive Experience | The AI made them feel good |
| Should Continue | Want them to continue, but difficult without support |
| Useful | AI is valued |
| Knowledge/Skills/Attitudes |  |
| Appreciating each other | HA’s feel more valued |
| Better understanding of each other | Know each other better |
| *Happier/Easier work* | Happier at work, workload shared between staff more |
| *Improved non-technical skills* | Teammates more flexible, better understanding of positives |
| Improved Knowledge | Better communication of abnormal observations |
| Improved resilience | Staff help each other to cope with the work e.g. all staff helping with pushing patients to theatre |
| Lobbying for change | Trying to get shoe rack, tapped buckets and getting help with traffic control from guards, trying to introduce rotas for CO’s |
| *Raising awareness to improve care* | More information signs for patients (visiting hours/hand washing) and staff (hand washing) |
| *Empowered, pride/respect in work* | HA’s feel empowered to change things, respect is increased at work |
| Behaviour Change |  |
| Altered interactions with staff/patients | Staff feel more able to approach seniors  Improved communication within team, addressing issues directly within the team (e.g. meeting with CO’s when they needed faster assessments of patients), improved teamwork across cadres, feel patients more open because staff attitude better. |
| Altered supervision/feedback methods | *** |
| *Individual altering behaviour* | More hand washing, CO’s more present on the wards |
| *Monitoring change* | Regular feedback to monitor changes |
| New forums to discuss ideas | New joint ward meetings, |
| Team alter way of working | Team remain at work to handover  PN & LW ward teams work better together, staff all emphasise hand washing, general surroundings of ward improved, Cleaning day planned, decided to redesign delivery packs with swabs inside them |
| Practice Changes/Patient Outcomes |  |
| *Patients changing behaviour* | Patients/guardians removal of shoes in KMC and Nursery |
| Development of protocols/guidelines/systems | Purchase of shoe rack,  Handovers became routine,  PN & LW take patients to theatre together  Health talk rotas about hand washing/traffic control, guards assigned to help with traffic control |
| Improved patient satisfaction | Feeling that reputation of hospital has improved |
| Improved retention/recruitment/sickness | *** |
| *Information shared with patients/relatives* | Health talks about hand washing and traffic control for infection prevention |
