# Supplementary material for: Assessing the impact of a motivational intervention to improve the working lives of maternity healthcare workers: a quantitative and qualitative evaluation of a feasibility study in Malawi
Source: Pilot Feasibility Stud. 2021 Jan 29;7:34. doi: 10.1186/s40814-021-00774-7 (PMC7844964; doi:10.1186/s40814-021-00774-7)
Supplement: Supplementary file 5 — Additional file 5. Qualitative summary from the Community Hospital. [file 40814_2021_774_MOESM5_ESM.docx]

| Kirkpatrick area & sub themes | Examples in the Community Hospital |
| --- | --- |
| Reaction | |
| Exciting/fun | Laughing about interviews, energisers fun |
| Positive Experience | Want to share skills in new departments, interactive and focusing on reinforcing good areas |
| Should Continue | Can't stop the changes need to continue them, benefiting the ward |
| Useful | Want to introduce AI to the hospital quality improvement team |
| Knowledge/Skills/Attitudes |  |
| Appreciating each other | HAs feel more appreciated as an active part of the team |
| Better understanding of each other | More accepting of people's suggestions because get to know each other in meetings |
| *Happier/Easier work* | Working more as a team so its easier, being able to depend on each other |
| *Improved non-technical skills* | Confidence, leadership, respecting patients more leading to better greeting of patients, facilitation, finding solutions |
| Improved Knowledge | Hand-washing, training HA’s to take observations, monitoring and examination of patients |
| Improved resilience | Building hand washing stations, help each other more if there are problems |
| Lobbying for change | More bins and personal protective wear on wards |
| *Raising awareness to improve care* | Hand-washing signs at sink, signs on bins, AI slogan use to secretly correct behaviour, talking to patients to give informal reminders |
| *Empowered, pride/respect in work* | Feel empowered after meetings, gain confidence as recognised and have more responsibility. |
| Behaviour Change |  |
| Altered interactions with staff/patients | Participants freely expressing themselves of all cadres, better relationships between nurses and clinicians, better communication with patients, can highlight mistakes by seniors now |
| Altered supervision/feedback methods | ** |
| *Individual altering behaviour* | hand washing, throwing waste in the correct bins, improved decontamination of instruments by COs, wearing closed shoes, fuller history and examinations |
| *Monitoring change* | monitoring tools developed and completed for action plans, accountability to the team |
| New forums to discuss ideas | ** |
| Team alter way of working | HA's start to take observations, incinerator better cared for, HAs meeting to discuss waste disposal, Hand-washing watch dogs, using AI slogan to remind team to wash hands, closed shoes monitoring, easier access to PPE, discussing AI tasks informally as well as in meetings |
| Practice Changes/Patient Outcomes |  |
| *Patients changing behaviour* | Hand-washing |
| Development of protocols/guidelines/systems | sharps boxes disposed of when ¾ full, rosters for patient talks developed |
| Improved patient satisfaction | *** |
| Improved retention/recruitment/sickness | Reduced sick leave |
| *Information shared with patients/relatives* | Health talks on hand-washing and waste disposal |
